# Electrophysical Properties of PMN-PT-Ferrite Ceramic Composites

**DOI:** 10.3390/ma12203281

**Published:** 2019-10-09

**Authors:** Dariusz Bochenek, Przemysław Niemiec, Ryszard Skulski, Dagmara Brzezińska

**Affiliations:** Faculty of Science and Technology, Institute of Materials Engineering, University of Silesia in Katowice, 75 Pułku Piechoty 1a, 41–500 Chorzów, Poland; przemyslaw.niemiec@us.edu.pl (P.N.); ryszard.skulski@us.edu.pl (R.S.); dagmara.brzezinska@us.edu.pl (D.B.)

**Keywords:** PMN-PT solid solutions, Ni-Zn ferrite, ceramic composites, multiferroics, micromechatronics

## Abstract

Ferroelectromagnetic composites based on (1−*x*)PMN-(*x*)PT (PMN-PT) powder and Ni-Zn ferrite powder were obtained and are described in this work. As a ferroelectric component, we used (1−*x*)PMN-(*x*)PT solid solution (with *x* = 0.25, 0.28, 0.31, 0.34, 0.37, 0.40), synthesized using the sol-gel method. As a magnetic component, we used nickel-zinc ferrite, obtained using classic ceramic technology. The six compositions of PMN-PT used have rhombohedral symmetry, tetragonal one and mixture of these phases (morphotropic phase area), depending on *x*. The final ceramic composite samples were obtained using the classic methods involving the calcination route and pressureless final sintering (densification). The properties of the obtained ceramic composite samples were investigated, including microstructure SEM (scanning electron microscope), dielectric properties, electromechanical properties, and DC (Direct Current) electrical conductivity. Results showed that the microstructures of the PP-F composite samples characterized by larger grains were better crystallized, compared with the microstructures of the PMN-PT ceramic samples. The magnetic properties do not depend on the ferroelectric component of the composite samples, while the insertion of ferrite into the PMN-PT compound reduces the values of remnant and spontaneous polarization, as well as the coercive field. The dielectric measurements also indicated that the magnetic subsystem influences the dielectric properties. The present results show that the PP-F ceramic composite has good dielectric, magnetic, and piezoelectric properties, which predisposes this type of material to specific applications in microelectronics and micromechatronics.

## 1. Introduction

In recent years, there has been a global search for multiferroics [1,2,3,4], magnetoelectric materials [5], materials with coupling between magnetic and polarization orders [6], materials with functional properties [7,8] also in the form of magnetoelectric composites [9,10,11,12,13], lead-free multiferroic composites [14,15], and multiferroic nanocomposites [16,17]. Many methods and techniques for obtaining ceramic powders are known, such as the solid state reaction method, two-stage columbite method [18], sol-gel method [19], coprecipitation method [20], mechanochemical activation [21], selective laser sintering (SLS) [22,23], self-propagating high-temperature synthesis (SHS) [24], gel combustion [25], and others. Each of these methods gives rise to new possibilities; however, at the same time, it requires the use of specific technological conditions and laboratory precision in order to obtain ceramic materials with optimal properties. The sol-gel technology has numerous advantages, e.g., lowers the temperature of the synthesis, provides perfect homogeneity of the powders of the multicomponent compounds, and does not require the use of complicated equipment. The two-phase multiferroic ceramic composites obtained by the authors and described in this paper consist of two components—ferroelectric/relaxor and ferromagnetic. As the ferroelectric/relaxor component, (1−*x*)PbMg_1/3_Nb_2/3_O_3_-*x*PbTiO_3_ (PMN-PT) was used, while nickel-zinc ferrite (Ni_0.64_Zn_0.36_Fe_2_O_4_) was used as the magnetic component.

PbMg_1/3_Nb_2/3_O_3_ (PMN) is a canonical relaxor with perovskite-type structure [26,27]. The temperature of the maximum dielectric permittivity of PMN is between −13 °C and −3 °C, depending on the frequency of measurements [28,29]. Of great practical importance are the solid solutions PMN-PT (i.e., (1−*x*)PbMg_1/3_Nb_2/3_O_3_-*x*PbTiO_3_ (abbreviated in this paper to PP)). In (1−*x*)PMN-*x*PT, the maximum dielectric permittivity shifts towards higher temperatures with increasing *x* (from *T_m_* = −3 °C for *x* = 0 to about *T_m_* = 227 °C for *x* = 0.5 [30,31]). The PMN-PT powders used by us were obtained using the sol-gel method, similar to the method described in Reference [32]. Nickel-zinc ferrite with a general formula Ni_1−*x*_Zn*_x_*Fe_2_O_4_ and spinel-type structure was used as a magnetic component. The composition used by the authors, i.e., Ni_0.64_Zn_0.36_Fe_2_O_4_, belongs to well-known soft ferrites with a high value of magnetic permeability and relatively high resistance *ρ* (10^5^ Ωm) [33], and was obtained using the classic ceramic method. The final composite samples were obtained using classic methods with calcination and pressureless final sintering (densification).

In previous work [34], the authors described ceramic composites based on two compositions of PMN-PT, i.e., 0.72PbMg_1/3_Nb_2/3_O_3_-0.28PbTiO_3_ with rhombohedral symmetry and 0.63PbMg_1/3_Nb_2/3_O_3_-0.37PbTiO_3_ with tetragonal symmetry. For the samples, X-ray Diffraction (XRD) patterns, SEM microstructure, energy dispersive spectrometer (EDS) spectra as well as dielectric, electrical, and magnetic properties were investigated. The obtained results presented in Reference [34] confirmed the existence of ferroelectric and ferrimagnetic properties at room temperature.

In previous work [35], the authors obtained and investigated composites based on six compositions of (1−*x*)PMN-*x*PT, with *x* = 0.25, 0.28, 0.31, 0.34, 0.37, 0.40. These compositions of PMN-PT have rhombohedral and tetragonal symmetry, as well as a mixture of these phases (morphotropic phase boundary (MPB)). In the composite samples obtained in such a way, XRD patterns, EDS spectra, as well as dielectric and magnetic properties were investigated [35]. The results confirmed ferroelectric and ferrimagnetic properties at room temperature in the obtained ceramic materials.

In the present work, the authors describe more detailed investigations of the electrophysical and magnetic properties as well as phase transitions of PMN-PT-ferrite ceramic composites with *x* = 0.25, 0.28, 0.31, 0.34, 0.37, 0.40 (i.e., the same as in Reference [26]). Inter alia, the following investigations were conducted: SEM microstructure, more detailed dielectric investigations, electromechanical properties, and DC (direct current) electrical conductivity.

## 2. Experiment

In this work, the ceramic samples of the PMN-PT solid solutions and the ceramic samples of the PP-F composites were obtained using the two-step columbite sol-gel technology, and were then compared. 

**Figure 1:**
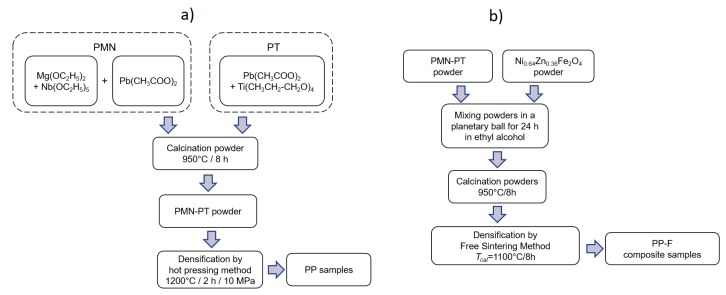
General scheme of technological process: (**a**) of the (1−*x*)PMN-*x*PT (PP) materials, (**b**) PMN-PT-ferrite (PP-F) composite samples.

The (1−*x*)PbMg_1/3_Nb_2/3_O_3_-(*x*)PbTiO_3_ (PP) powders were obtained with the sol-gel technology, using two-step columbite method. In the first step, the PbMg_1/3_Nb_2/3_O_3_ component (PMN) was obtained as a result of the reaction between Mg(OC_2_H_5_)_2_ and Nb(OC_2_H_5_)_5_ in ethyl alcohol, while in the second step, Pb(CH_3_COO)_2_ and ethylene glycol were added to the mixture. The PbTiO_3_ component (PT) was obtained as a result of the reaction between Pb(CH_3_COO)_2_ and Ti(CH_3_CH_2_-CH_2_O)_4_. The liquid compositions PMN and PT were mixed together in the proper proportions with *x* = 0.25, 0.28, 0.31, 0.34, 0.37, 0.40. After drying the gel to a powder, the sintering (densification) of the PMN-PT samples was conducted by hot uniaxial pressing at the following conduction: *T_s_* = 1200 °C, *t_s_* = 2 h, *p_s_* = 10 MPa. The technology for obtaining the compositions was described in detail in Reference [22]. The powder of Ni_0.64_Zn_0.36_Fe_2_O_4_ ferrite was obtained from simple oxides NiO (Sigma-Aldrich, St. Louis, MO, USA, 99.8% purity, <50 nm particle size), Fe_2_O_3_ (Sigma-Aldrich 99.98% purity, <5 μm particle size) and ZnO_2_ (POCH, Gliwice, Poland, 99.9% purity, <45 μm particle size), using the calcination technique under the conditions of 1100 °C/4 h. The general scheme of the technological process of the PP ceramic samples is presented in Figure 1a. 

The designed composites, based on six compositions of PMN-PT (with *x* from 0.25 to 0.40) and Ni_0.64_Zn_0.36_Fe_2_O_4_ powder, consisted of 90 wt-% of PMN-PT type ceramic powder and 10 wt-% of ferrite powder. The powders of each composition were mixed using a Fritsch planetary ball mill for 15 h (wet in ethyl alcohol), and the mixture of powders was then calcined in following conditions: *T_calc_* = 950 °C and *t_calc_* = 8 h. The compacting (densification) of the composite ceramic samples was conducted at a conduction of *T_s_* = 1100 °C by *t_s_* = 8 h. Afterwards, the ceramic samples were grinded, polished, and annealed at 700 °C in order to remove mechanical stresses. The final step of the technological process involved applying the silver paste electrodes onto both surfaces of the specimens for electrical testing. The technology of obtaining the composite compositions was described in detail in Reference [34]. The general scheme of the technological process of the PP-F ceramic composite samples is presented in Figure 1b.

The designed compositions of the PMN-PT-ferrite (PP-F) with the general formula 0.9[(1−*x*)PMN-*x*PT]-0.1(Ni_0.64_Zn_0.36_Fe_2_O_4_) with *x* = 0.25, 0.28, 0.31, 0.34, 0.37, 0.40, were identified as PP25-F, PP28-F, PP31-F, PP34-F, PP37-F, PP40-F, respectively. 

The microstructure tests of the composite samples were performed using a JEOL (Tokyo, Japan) JSM-7100 TTL LV field emission scanning electron microscope equipped with an energy dispersive spectrometer (EDS) and a backscattered electron detector (BSE). The dielectric measurements (in a temperature range from 20 °C to 450 °C) were performed using a capacity bridge of a QuadTech 1920 LCR meter (Quad/Tech, Inc., Maynard, MA, USA) for the cycle of heating (at frequencies of the measurement field from 1 kHz to 100 kHz). The hysteresis (*P–E*) loops were investigated with a Sawyer-Tower circuit and a Matsusada Inc. (Kusatsu, Japan) HEOPS-5B6 high voltage amplifier. The electromechanical measurements were carried out using an optical displacement meter (Philtec Inc., Annapolis, MD, USA, D63) and a HEOPS-5B6 high voltage amplifier. The data were stored on a computer disc using an A/D, D/A transducer card and the LabView computer program. The DC electrical conductivity was measured using a Keithley 6517B electrometer high-resistance meter (Cleveland, OH, USA) in a temperature range from 20 °C to 480 °C.

## 3. Results and Discussion

In previous work [35], the XRD measurements of the six compositions of (1−*x*)PMN-*x*PT with *x* = 0.25, 0.28, 0.31, 0.34, 0.37, 0.40, as well as the ceramic composite PP-F obtained based on them, were presented. The PMN-PT has rhombohedral symmetry, tetragonal symmetry, and a mixture of these two phases (morphotropic area) that determine the electrophysical properties of the materials. The XRD (PANalytical, Phillips X’Pert Pro, Eindhoven, The Netherlands) tests of the composite PP-F samples additionally showed small peaks derived from the ferrite phase (cubic spinel lines). Moreover, the EDS tests (not presented here) confirmed the assumed chemical composition of PP-F and the presence of maxima from elements originating in PMN-PT, as well as elements originating in ferrite [35].

**Figure 2:**
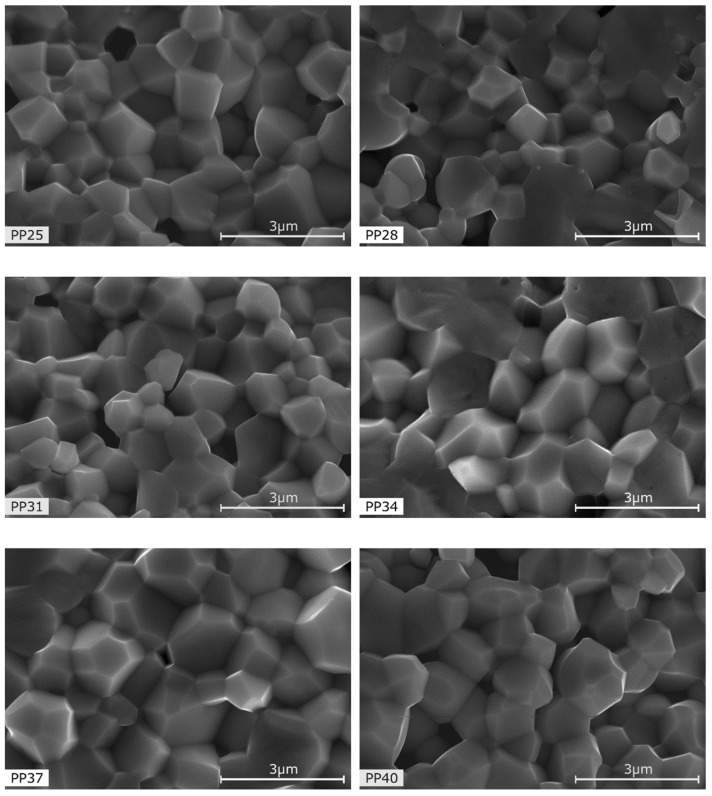
SEM microstructure of PP ceramics for: PP25 for *x* = 0.25, PP28 for *x* = 0.28, PP31 for *x* = 0.31, PP34 for *x* = 0.34, PP37 for *x* = 0.37, PP40 for *x* = 0.40.

Figure 2 shows the microstructural SEM images of the fractured PMN-PT ceramic samples (PP) obtained using the sol-gel method. The PP samples with rhombohedral symmetry (Figure 2—PP25, PP28) have a fine-grained microstructure, with well-crystallized grains. On the other hand, in the PP samples with tetragonal symmetry (Figure 2—PP37, PP40), slightly larger grains are observed. In the PP samples from the morphotropic area both small and large grains are present (Figure 2—PP31, PP34).

**Figure 3:**
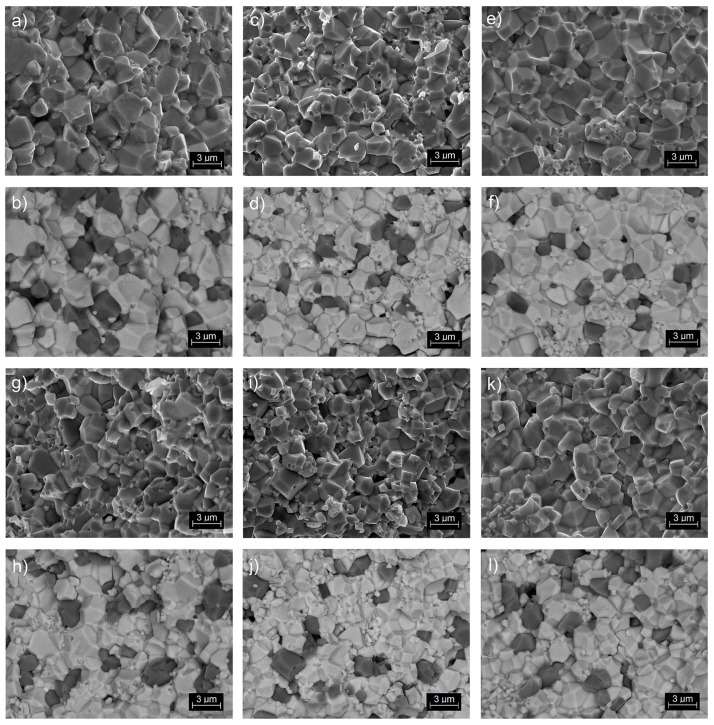
SEM microstructure of PP-F samples: (**a**,**b**) PP25-F, (**c**,**d**) PP28-F, (**e**,**f**) PP31-F, (**g**,**h**) PP34-F, (**i**,**j**) PP37-F, (**k**,**l**) PP40-F. The images were taken in the standard SB mode (detection of the assembly of signals from the secondary and backscattered electron detectors—Figure 3a,c,e,g,i,j) and BSE (detection of backscattered electrons—Figure 3b,d,f,h,j,l).

Figure 3 shows microstructural SEM images of the PMN-PT-ferrite composite samples (PP-F). The images were taken in the standard SB mode (detection of the assembly of signals from the secondary and backscattered electron detectors—Figure 3a,c,e,g,i,k), as well as BSE mode (detection of backscattered electrons—Figure 3b,d,f,h,j,l). In the BSE mode, the backscattered electrons are predominantly detected thanks to the filtering of low-energy secondary electrons. The observation of backscattered electrons allows the visualization of differences in the composition of the sample using different levels of contrast. Using a reduced signal level from the backscattered electron detector, one can observe an image of the composition of elements with a higher atomic number (bright area) and the topography of the elements with a lower atomic number (dark area), e.g., ferrite grains. 

The BSE images of the microstructure of fractures obtained for the PMN-PT-ferrite composites show the existence of a PMN-PT matrix (which is of higher atomic number, appearing white) and ferrite inclusions (dark grains with lower atomic number). The SEM tests confirmed that the ferrite grains are randomly distributed throughout the entire sample volume. Comparing the microstructures from Figure 2 and Figure 3, one can see that in PP-F samples, the grains are larger and better crystallized. Just as in the PP samples, in the PP-F samples with compositions from the MPB area both small and large grains are visible. 

Previous magnetic measurements [35] confirmed that above room temperature, the obtained composite materials exhibit magnetic (ferrimagnetic) properties, with very slim magnetic hysteresis loops, and that such magnetic properties do not depend on the ferroelectric component of the composites. 

**Figure 4:**
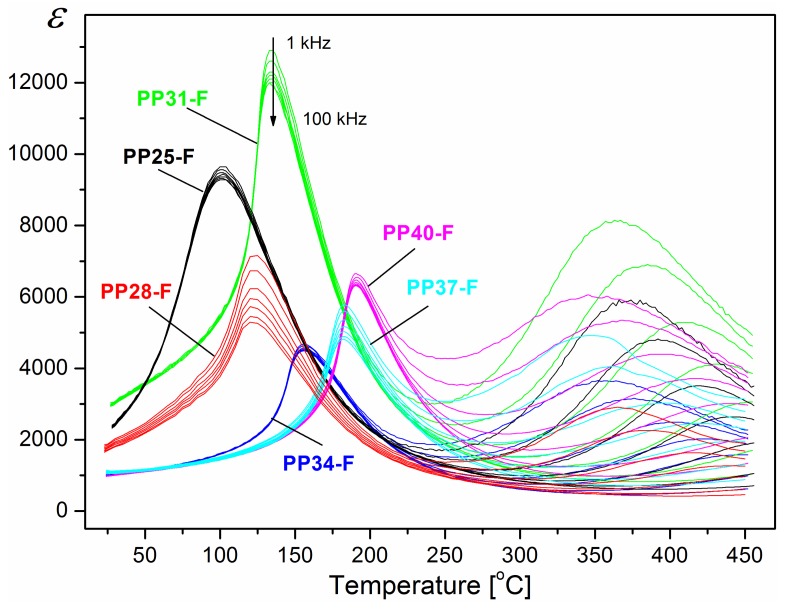
Temperature dependencies of dielectric permittivity *ε* for the PP-F ceramic composites (heating cycle, frequency: 1 kHz, 2 kHz, 5 kHz, 10 kHz, 20 kHz, 50 kHz, 100 kHz).

Figure 4 compares the temperature dependence of dielectric permittivity for the PP ceramics and the PP-F composite samples obtained at various frequencies. In the case of the PP-F, the maximum dielectric permittivity occurs in a narrower range of temperatures. The phase transition does not depend on the frequency of the measuring field. Analysing *ε*(*T*) dependencies above the ferroelectric-paraelectric phase transitions, one can observe the next maximum with a very broad and dispersive peak of dielectric permittivity for all investigated PP-F composite samples. One reason for this may be the presence of the magnetic component (ferrite) in the composite exhibiting phase transition in this area. In the same range of temperatures, a strong frequency dependency is observed (Figure 4) for the obtained compositions. These broad peaks shift towards higher temperatures with increasing frequency of the measurement field. This phenomenon may be associated with the imposition of a number of factors, for instance, as a result of relaxation processes as well as an increase in electrical conductivity in composite samples at high temperatures [36,37,38,39,40]. 

**Figure 5:**
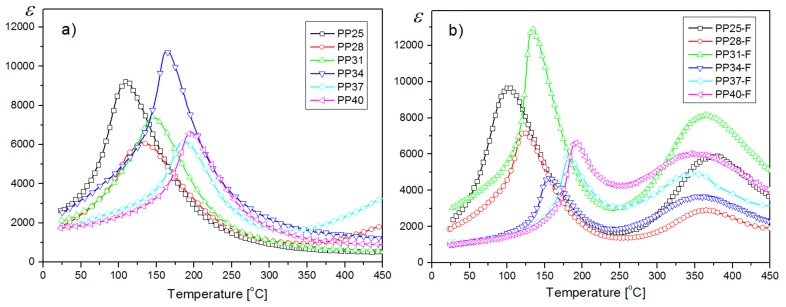
Temperature dependencies of dielectric permittivity for PP ceramics (**a**) and for PP-F ceramic composites (**b**) (heating cycle, frequency 1 kHz).

**Figure 6:**
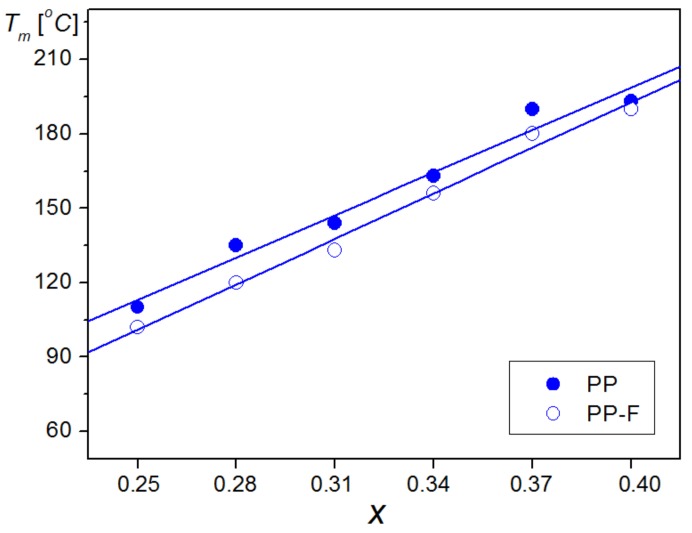
Dependencies of temperature of maximum dielectric permittivity *T_m_*(*x*) on *x* (heating cycle, frequency 1 kHz).

For a clear comparison, the results of the dielectric investigations of samples are presented in Figure 5a (for PP samples) and Figure 5b (for PP-F composite samples) for 1 kHz frequency. In the majority of investigated compositions of PP-F, the values of maximum dielectric permittivity are higher in comparison with the PP samples. It is also seen that for PP-F samples, the temperatures at which the maxima of dielectric permittivity take place (*T_m_*) are shifted towards lower temperatures in comparison with the PP samples (Figure 6, Table 1 and Table 2).

**Figure 7:**
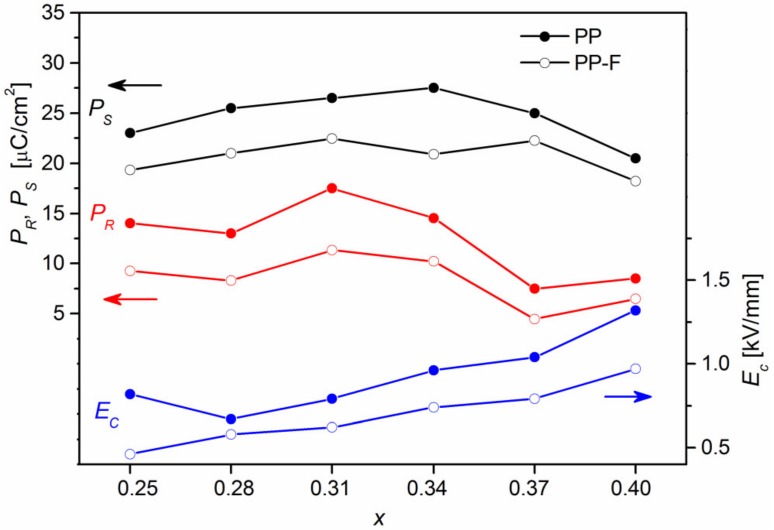
Dependence of remnant polarization *P_R_*, spontaneous polarization *P_S_*, and coercive field *E_c_* of the (1−*x*)PMN-*x*PT with *x* = 0.25, 0.28, 0.31, 0.34, 0.37, 0.40.

The measurement of *P(E*) electric hysteresis loops for PMN-PT solid solutions and PP-F composite materials showed that increasing the amount of PT in PMN-PT causes an increase in the value of the coercive field in the PP-F composite samples (Figure 7). The composite samples with tetragonal structures exhibit lower values of spontaneous polarization (*P_S_*) and remnant polarization (*P_R_*), while the samples from the MPB morphotropy area are characterized by indirect values of coercive field (*E_c_*), *P_R_*, and *P_S_*. The obtained results confirm ferroelectric and weak ferromagnetic properties at room temperature for the obtained ceramic composite samples. The *P*(*E*) electric hysteresis loops at room temperature and frequency of 1 Hz for the obtained materials were presented in Reference [26]. 

**Figure 8:**
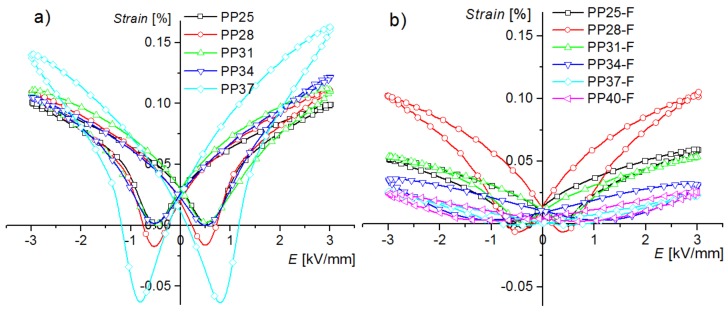
Bipolar strain-electric field loops (*S*–*E*) for (**a**) PP ceramics and (**b**) PP-F composite materials.

The electromechanical properties of the PMN-PT solid solutions and the ceramic PP-F composites are presented in Figure 8a,b, respectively. The measurements show that the strains in the samples with ferrite are lower. This could be expected, since part of the volume is filled with material that does not exhibit piezoelectric/electrostrictive properties. The *S-E* electromechanic loops have a specific shape (“butterfly wings”). For the majority of the investigated samples, in some range of *E,* a nearly linear relation *S*(*E*) is observed, which indicates the piezoelectric nature of the strain. The values of *S_rest_* residual strain and *H_S_* strain hysteresis coefficient for PMN-PT ceramic samples demonstrate higher values compared with the composite PP-F samples (Table 1 and Table 2). The strain hysteresis coefficient *H_S_* was calculated using the following formula [41]:

(1) $$H_{S}=\frac{{\Delta S}_{half}\times 100\%}{S_{max}}$$

where Δ*S_half_* is the hysteresis of strains (the difference between maximum and minimum strain value for the half of the maximum electric field) and *S_max_* is the strain value for the maximum of the applied electric field. 

**Figure 9:**
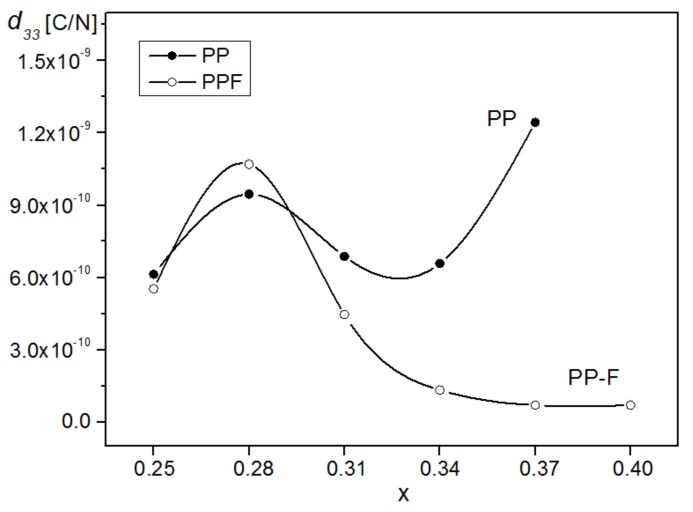
Dependencies of the piezoelectric coefficient *d_33_* of the obtained ceramic samples (frequency 0.1 Hz).

The piezoelectric coefficient of the obtained ceramic samples measured using a frequency of 0.1 Hz and calculated as a maximal value of the derivative $\frac{\partial S}{\partial E}$ is presented in Figure 9, as well as in Table 1 and Table 2, for the PP samples and the PP-F composites, respectively. In the great majority of compounds, much higher values of the piezoelectric properties are observed for the PMN-PT solid solutions. The PMN-PT single crystals with PT content from 27% to about 33% have superior piezoelectric properties in relation to the traditional PZT ceramics [42,43,44]. For the PMN-28PT, the ceramics deformation is slightly smaller than in classic PZT piezoceramics (e.g., PZT-5H ceramics), but still has a high strain value.

For the obtained ceramic samples of PMN-PT, the highest values of the piezoelectric coefficient (*d_33_*) are exhibited by the samples with *x* = 0.37 (tetragonal phase) and *x* = 0.28 (rhombohedral phase). Introduction to PMN-PT compounds of the ferrite compound significantly reduces the value of *d_33_* (except for the PP28-F composition). The highest value of *d_33_* is exhibited by the composition with *x* = 0.28, and the successive values of *d_33_* decrease with increasing *x* in PMN-PT in PP-F composite samples.

**Figure 10:**
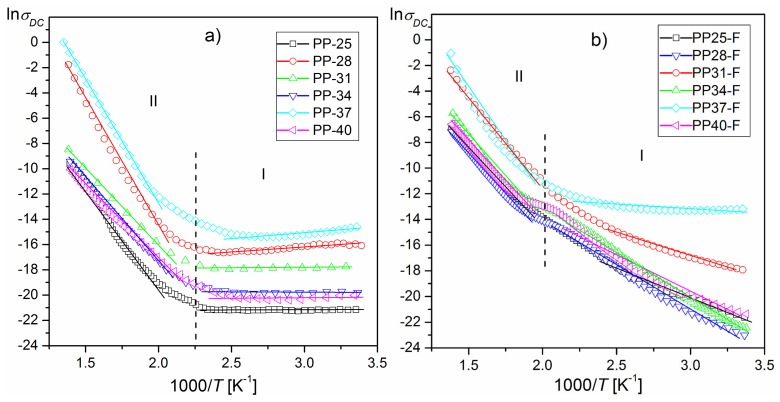
Influence of *x* (PT) in PP ceramics (**a**) and PP-F composite samples (**b**) on ln*σ_DC_*(1000/*T*) dependencies.

The results of the investigations of DC electrical conductivity are presented in Figure 10a (for PP) and Figure 10b (for PP-F). Quite significant differences in dependency ln*σ_DC_*(1000/*T*) (consisting of an increase in electrical conductivity) between the PP and PP-F samples are observed for 1000/*T*, in the temperature range 330–500 K (57–227 °C), which denotes significant differences in electrical conductivity near the phase-transition temperature. These differences are more pronounced for the samples with less content of PbTiO_3_. This can be important information in the development of technologies of composite materials. 

The graphs ln*σ_DC_*(1000/*T*) show changes in the slope of the curves (two regions of the graph) with different values of activation energy. Activation energy was calculated from the slope of the ln*σ*_DC_(1000/*T*) plot according to the Arrhenius law [45]:

(2) $$\sigma_{DC}=\sigma_{0}e^{-\frac{E_{Act}}{k_{B}T}}$$

where *σ_0_* is the pre-exponential factor, *k_B_* is the Boltzmann constant, *E_Act_* is the activation energy, and *T* is the absolute temperature. Values of the activation energy calculated according to Formula (2) in two temperature areas are given in Table 1 and Table 2.

## 4. Conclusions

New ferroelectromagnetic ceramic PP-F composites based on PMN-PT and ferrite powders, as well as PMN-PT ceramic samples, were successfully obtained and compared. The PP samples with rhombohedral symmetry have a fine-grained microstructure, with well-crystallized grains, while the PP samples with tetragonal symmetry have rather larger grains. The microstructure of the PP samples from the MPB morphotropic area shows both small and large grains. The microstructures of the PP-F composite samples characterised by larger grains are better crystallized.

Previous magnetic measurements confirmed that above room temperature, the obtained composite materials show magnetic (ferrimagnetic) and ferroelectric properties, and that the magnetic properties do not dependent on the ferroelectric component of the composites. Addition of ferrite to the PMN-PT compound leads to a decrease in the values of remnant and spontaneous polarization, and coercive field.

The dielectric measurements show that in all PP-F samples, an additional maximum of dielectric permittivity at temperatures about 350–370 °C is observed, which does not exhibit the dispersion of dielectric permittivity in the frequency range of 10^3^–10^5^ Hz. The dielectric measurements indicated that the magnetic subsystem influences the dielectric properties (value of dielectric permittivity and dielectric loss). 

The designed and obtained ceramic PP-F composite with good dielectric, magnetic, and piezoelectric properties confirmed the adequately conducted ceramic technology (i.e., synthesis by sol-gel method of the PMN-PT powders, selection of the sintering conditions, appropriate proportion of ferrite and ferroelectric powder, etc.), which allows the production of ferro-electro-magnetic materials with very interesting properties for specific applications in microelectronics and micromechatronics. These types of materials are very suitable for use in various types of piezoelectric transducers, sensors, servomotors, phase modulators, magnetoelectric transducers, piezoelectric-magnetostrictive accelerometers, etc.

## Figures and Tables

**Table 1:** Electrophysical parameters of PMN-PT solid solutions.

Parameter	PP25	PP28	PP31	PP34	PP37	PP40
*ρ* [g/cm^3^]	7.15	7.45	7.35	7.51	7.21	7.01
*T_m_* [°C]	110	132	146	163	187	195
*ε_r_*	2620	1730	2080	2390	1830	1700
*ε_m_*	9170	6050	7350	10,760	6010	6620
tan*δ* at *T*	0.113	0.023	0.092	0.200	0.021	0.118
tan*δ* at *T_m_*	0.108	0.023	0.062	0.225	0.030	0.101
*P_S_* [µC/cm^2^]	22.30	24.86	25.70	27.30	25.70	20.16
*P_R_* [µC/cm^2^]	19.31	21.00	22.46	20.90	22.26	18.22
*E_c_* [kV/mm]	0.82	0.67	0.79	0.96	1.04	1.32
*E_Act_* in I [eV]	0.009	0.059	0.011	0.012	0.052	0.017
*E_Act_* in II [eV]	1.412	1.729	0.996	1.069	1.619	0.936
*H_S_* [%]	13.61	14.06	29.52	12.20	37.51	-
*S_rest_* [%]	0.029	0.020	0.029	0.024	0.017	-
*d_33_* [pC/N]	613	946	956	687	1243	-

*ρ—*density*, T_m_—*temperature at maximum of dielectric permittivity, *ε_r_*, *ε_m_—*dielectric permittivity at room temperature and *T_m_*, respectively, tan*δ—*dielectric loss, *P_S_*, *P_R_—*spontaneous and remnant polarization, respectively, *E_c_—*coercive field*, E_Act_—*activation energy, *H_S_—*strain hysteresis coefficient, *S_rest_—*residual strain, *d_33_—*piezoelectric coefficient.

**Table 2:** Electrophysical parameters of PP–F composite materials.

Parameter	PP25-F	PP28-F	PP31-F	PP34-F	PP37-F	PP40-F
*ρ* [g/cm^3^]	7.20	7.41	7.39	7.13	7.19	7.02
*T_m_* [°C]	102	123	134	157	182	191
*ε_r_*	1860	1750	2900	1090	1225	980
*ε_m_*	9660	7180	12,910	4750	9910	6630
tan*δ* at *T_r_*	0.007	0.040	0.009	0.005	0.008	0.007
tan*δ* at *T_m_*	0.026	0.137	0.059	0.041	0.063	0.061
*P_S_* [µC/cm^2^]	13.21	12.02	16.57	13.90	7.58	9.29
*P_R_* [µC/cm^2^]	9.26	8.30	11.32	10.23	4.45	6.46
*E_c_* [kV/mm]	0.46	0.58	0.62	0.74	0.79	0.97
*E_Act_* in I [eV]	0.377	0.346	0.623	0.610	0.062	0.438
*E_Act_* in II [eV]	0.978	1.147	1.287	1.130	1.483	1.109
*H_S_* [%]	15.45	18.28	7.32	63.30	19.16	38.51
*S_rest_* [%]	0.012	0.014	0.011	0.010	0.001	0.004
*d_33_* [pC/N]	553	1070	446	133	70	71
